# Chloride Penetration of Surface-Coated Concrete: Review and Outlook

**DOI:** 10.3390/ma17164121

**Published:** 2024-08-20

**Authors:** Jing Liao, Yuchi Wang, Xiping Sun, Yuanzhan Wang

**Affiliations:** 1 Simulation State Key Laboratory of Hydraulic Engineering Intelligent Construction and Operation, Tianjin University, 135 Yaguan Road, Jinnan District, Tianjin 300072, China; liaojing@tju.edu.cn (J.L.); yzwang@tju.edu.cn (Y.W.); 2 Tianjin Research Institute for Water Transport Engineering, M.O.T., 2618 Xingang 2nd Road, Binhai New District, Tianjin 300000, China

**Keywords:** concrete coating, chloride resistance, improvement efficiency, silane coating, nanomaterial-modified coating, organic–inorganic composite coating

## Abstract

Concrete coatings show significant promise in shielding concrete substrates from corrosion by effectively resisting harmful ions and moisture. Thanks to their practicality, high efficiency, and cost-effectiveness, coatings are considered a potent technique for enhancing the chloride resistance of reinforced concrete structures. Over recent decades, extensive research has concentrated on employing coatings to bolster concrete’s ability to withstand chloride penetration. This paper provides a holistic review of the current studies on chloride infiltration in concrete surfaces treated with coating materials, primarily focused on chloride resistance improvement efficiency and chloride transport modeling. Firstly, by comparing the functions of assorted coatings, four inherent protection mechanisms are summarized and elaborated thoroughly. Afterwards, the chloride resistance improvement efficiency of assorted coatings reported in current studies are reviewed and compared in great detail, with a specific focus on inorganic, organic, and organic–inorganic composite coatings. Furthermore, the theoretical research about methodologies for chloride transport behavior prediction is summarized. Finally, this paper outlines the potential research directions in this field and the theoretical, technical, and practical application challenges. This review not only identifies critical areas necessitating further investigation and problem-solving in this domain but also aids in selecting appropriate coating materials and refining corrosion management strategies.

## 1. Introduction

Concrete, a bulk construction material, plays an important role in modern infrastructure due to its prominent merits in terms of availability, moldability, compressive strength, durability, and cost [1]. Nevertheless, reinforced concrete (RC) structures in the marine environment may experience durability failure due to corrosion issues caused by chloride attack, which may trigger safety failure, demand repair or reestablishment, and bring economic losses and over-consumption of natural non-renewable resources [2]. For example, it is reported that the United Arab Emirates (UAE) spent about 5.2% GDP on repair or rehabilitation of RC structures during 2009–2011 [3]. This reminds stakeholders to deal with durability issues with discretion. There is an array of strategies available to prevent steel corrosion and enhance the longevity of RC structures, such as utilizing more durable cement material, incorporating corrosion inhibitors in concrete mixtures, applying protective coatings to the concrete surface, using stainless steel reinforcement or galvanized reinforcement, implementing cathodic protection systems, etc. Among these strategies, surface coating stands out due to its affordability, simplicity in application, and robust protective capabilities, and can be utilized during construction or service.

During the last few decades, the effect of concrete surface coating on chloride penetration has received increasing attention. For example, Saricimen et al. [4] analyzed the effect of a cementitious material coating, a mixture of Portland cement, fine silica, and an active chemical agent on the chloride resistance of in situ RC structures. The obtained chloride profiles of all components showed that chloride content decreased after the application of Portland cement coating. Moon et al. [5] investigated the chloride resistance of calcium–silicate compound-coated concrete using the steady-state chloride migration test. The results indicated the coated concrete had almost an 80% lower effective chloride diffusion coefficient compared with the control group. Medeiros and Helene [6] investigated and compared the chloride diffusion coefficients of concrete coated with a silane–siloxane mixture dispersed in water, silane–siloxane mixture dispersed in solvent, acrylic dispersed in solvent, and polyurethane. The results indicated that the aforementioned coating systems reduced the chloride diffusion coefficients by 9%, 17%, 20%, and 86%, respectively. This indicated the conventional organic coating polyurethane showed the most effective protection against chloride erosion. However, as is well known, conventional organic coatings represented by polyurethane have fatal defects of environmental pollution [7]. Kong et al. [8] successfully fabricated a superhydrophobic concrete coating (with a static contact angle of 159.1 degrees) into a concrete substrate. A non-toxic micro-diatomaceous earth, nano-Al_2_O_3_, and low-energy material were combined to fabricate a superhydrophobic coating with a micro–nanostructure. This study underscored the efficacy of protecting concrete against chloride erosion based on dynamic potential polarization curve measurement. Likewise, Liao et al. [9] put forward a hydrophobic nanocomposite coating fabricated by the combination of tannic acid, aminopropyl triethoxysilane, and a low-energy compound, hexadecyltrimethoxysilane (C_19_H_41_O_3_Si). They investigated the effect of concentration mix ratio on coating performance and advised the best concentration combination. They also claimed superiority of this coating in economic performance by comparison with epoxy resin coating. Li et al. [10] investigated the effect of coating category on the instantaneous and long-term performance of coated concrete. They stated that although the coated concrete with organic coating showed superior improvement in instantaneous chloride resistance characterized by passing coulomb electric flux compared to an infiltrating coating (silane-based coating), the latter showed slower aging under similar climate exposure conditions and higher chloride resistance in the long run. This can be attributed to the difference in protection mechanisms of these two kinds of coating.

To conclude, with the efforts of the research community, many concrete surface-coating materials are presently available to apply. And studies to date have shown encouraging results on the improvement effect of the surface coatings on chloride resistance, a crucial indicator of coating efficacy. The coating category, protection mechanism, fabrication method, etc. affect the performance of coated concrete in chloride resistance (instantaneous or long term). And different coatings perform differently in economic and environmental respects. Therefore, a comprehensive review of current research on chloride penetration in surface-coated concrete is necessary for providing a comprehensive evaluation of these techniques and guidance for the design and development of new techniques.

This study aims to present a comprehensive review of chloride penetration in coated concrete, focusing on coating protection mechanisms, improvement efficacy, chloride transport modeling, etc. Economic and environmental performance, as well as the influence of construction technology, are also considered, but are not extensively elaborated upon. Following this introduction, improvement mechanisms for coating on chloride resistance of concrete are first discussed in Section 2, serving as the basis for the review of the studies on chloride resistance of coated concrete in Section 3, focusing on inorganic and organic coatings. Research into the modeling of chloride transport behavior in coated concrete is outlined in Section 4. Finally, Section 5 presents an outlook, outlining challenges and offering recommendations for future research directions, with the aim of promoting practical engineering applications.

## 2. Protection Mechanism of Surface Coating

A comprehensive understanding of the protection mechanism of surface coating against chloride erosion is crucial for evaluating the improvement efficacy of current techniques in chloride resistance and optimizing their structures and composition to achieve better performance and characteristics. In this section, studies into the chloride erosion mechanism in concrete and several protection mechanisms of different coating types are reviewed, based on which the measurement methods for chloride resistance of surface-coated concrete are summarized.

### 2.1. Chloride Erosion Mechanism in Concrete

Under normal circumstances, the pH value of the concrete pore solution is above 12 [11], which is in an alkaline state. And the surface of the embedded steel bar is in a passivation state in an alkaline state, which does not rust easily [12,13]. With the chloride erosion and the increase in the surrounding chloride concentration, the pH value decreases, which leads to the corrosion of the steel bar, and ultimately leads to damage to and performance decline of the concrete structure. There are two main chloride transport mechanisms considered in this study: (i) chloride ion diffusion in the pore solution driven by concentration gradient: the external chloride will diffuse into the concrete as ions along the pore solution under the concentration gradient during long-term service in high-salinity environments, such as marine environments or road salts with high chloride content used for icing prevention; and (ii) penetration of water carrying chloride ions: the external solution with high chloride concentration will penetrate into the pores of concrete, causing a chloride concentration change. In addition, the chloride transport process is also affected by electromigration, physiochemical adsorption, etc. which are not the focus of this review.

### 2.2. Improvement Mechanism of Surface Coating on Chloride Resistance of Concrete

The protective mechanism is intricately tied to the specific coating type, with varying coatings offering protection via distinct mechanisms.

**Figure 1:**
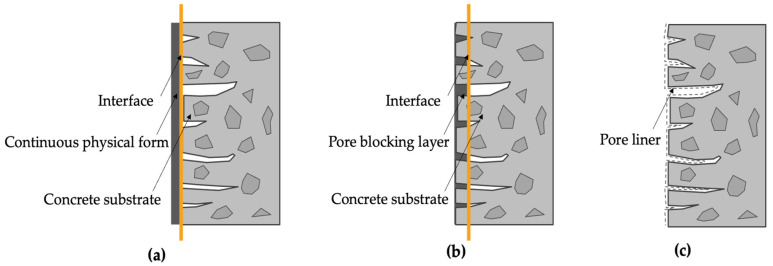
Types of surface coating-protection mechanisms: (**a**) film forming; (**b**) pore blocking; (**c**) pore liner.

According to [6,14], three fundamental protection mechanisms are identified for coating materials: (i) forming a continuous physical film on the original concrete surface (Figure 1a); (ii) reacting with certain cementitious material constituents to generate non-soluble fillers in the pores and cracks of concrete (Figure 1b); and (iii) forming a continuous waterproof layer on the concrete surface (Figure 1c). It is worth noting that a particular coating may correspond to a single protection mechanism or it may have multiple protection mechanisms acting simultaneously.

When the film is denser than the original concrete structure, it can play a protective role. The most common film-forming coatings are polymer coatings, such as acrylic emulsion coatings, epoxy coatings, polyurethane coatings, and fluorocarbon coatings, etc., which form a uniform film after application through a chemical reaction or physical process that covers the surface of the substrate. In the study of Barbucci et al. [15], the water permeability coefficients of a conventional organic polymer coating coated concrete were decreased by 72–95% when the film thickness was 0.02–0.04 cm. It has been stated that the adhesion and cracking bridging capacity of polymer coating are excellent [16], although the aging problem is prominent.

Certain coatings can react chemically with the surface of the concrete and form substances that fill the pores of the concrete, such as silicate gels. It is reported that the ethyl silicate can react with water and calcium hydroxide to generate non-soluble fillers, which means they serve as a kind of penetrant [17]. In addition, ethyl silicate can also form a layer of silicate film to play a protective role when coated on the surface of the concrete. The coatings act as both film protection and penetrant can as sealant [17]. Similar coatings include alkali-activated cement coatings [18]. When the cement in concrete reacts with water, it releases basic ions, such as hydroxide ions (OH^−^) and calcium hydroxide (Ca(OH)_2_). These alkaline substances react with carbon dioxide in the air on the concrete surface to form carbonates. This process is called alkali excitation. The formation of carbonate helps to fill the pores of concrete, reduce the penetration of water and harmful substances, and improve the compactness and durability of concrete [18].

The typical pore liner coatings, i.e., silane-based water repellent agents, can form a continuous waterproof layer on the concrete surface. The silane-based water repellent agents penetrate into the micropores and capillary pores in the concrete and form hydrophilic silyl compounds. These compounds fill the microscopic pores of the concrete, reducing the ability of water and dissolved salts to enter the interior of the concrete, thereby reducing the seepage properties of the concrete [19]. Silane coatings have excellent weather resistance and can resist the influence of natural environmental factors such as ultraviolet radiation, high temperature, and humidity changes. These characteristics make the coating not susceptible to aging, discoloration or failure, and maintain a long-term stable protective effect. In [20], it was suggested that silane-based coating offered a residual water protection effect even after 20 years of service, with a confidence level of 97%.

**Figure 2:**
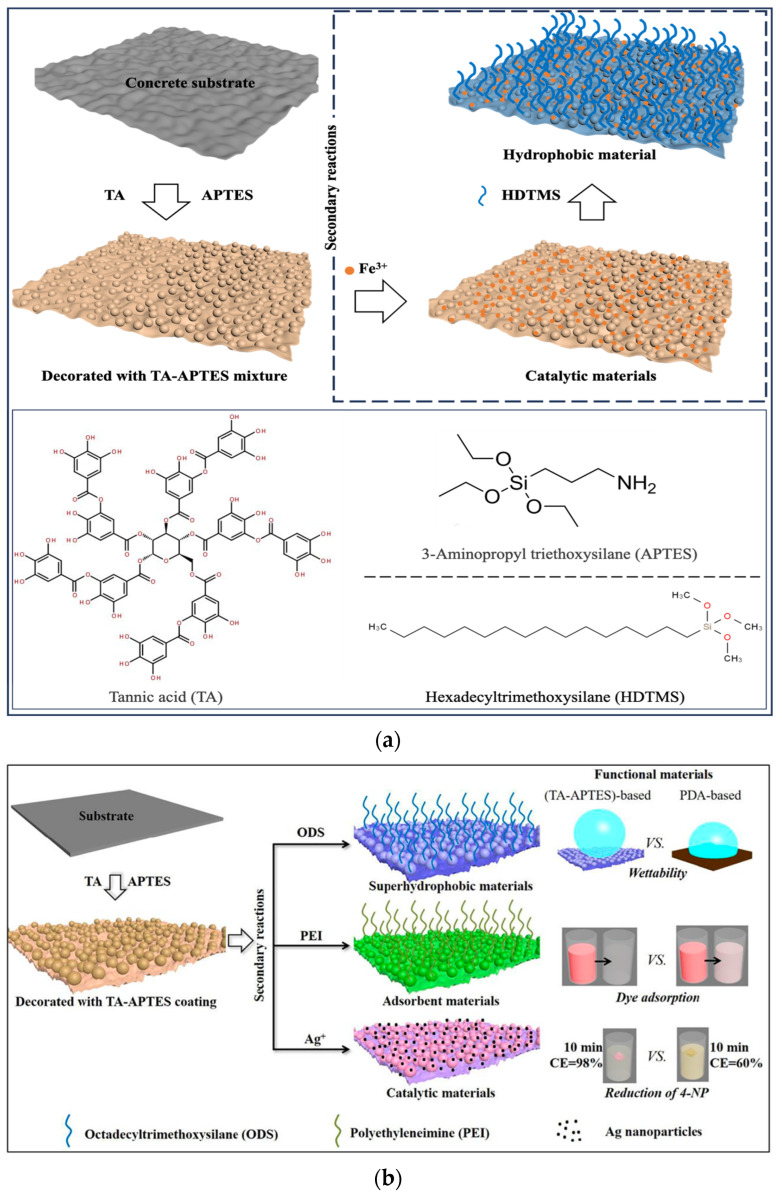
(**a**) Schematic illustration of the hydrophobicity coatings modified by hexadecyltrimethoxysilane [9]; (**b**) schematic illustration of the hydrophobicity coatings modified by octadecyltrimethoxysilane [25].

With the continuous progress in the field of coatings, additives and materials science, as well as people’s concern for environmental protection and sustainable development, scientists’ research investment in waterproof materials and technologies is increasing, and continuous innovation in materials, processes and construction technologies has promoted the development and application of the new technology of hydrophobic composite concrete coating (Figure 2a). The new hydrophobic coating constructs a hydrophobic layer on the surface of the concrete, reducing the possibility of surface erosion by water. A common practice is to construct composite coatings using nanomaterials to modify the coating substrates composed of low-surface-energy substances [21]. Currently, the primary coating substrates include polymers, copolymers, and polymer blends [22]. Common nanomaterials are silica, titanium dioxide, graphene oxide and so on. Mechanical mixing or ultrasonic dispersion can be used to break the van der Waals forces between nanoparticles [23]. Silane coupling agents and surfactants can be used to graft functional groups on the surface of nanoparticles to achieve chemical dispersion [24]. This dispersion of nanoparticles allows the coated surface to reach or approach a superhydrophobic surface, with a water contact angle of about 150 degrees [25], as shown in Figure 2b.

### 2.3. Measurement Methods for Chloride Resistance of Concrete

The methods for measuring the resistance of concrete to chlorine erosion mainly include the determination of chloride diffusion coefficient and chemical analysis. The former includes the standardized steady-state migration method according to NT Build 355 [26], the non-steady-state chloride migration test according to NT Build 492 [27] and the rapid chloride permeability test according to ASTM C1202/97 [28]. It worth noting that the rapid chloride permeability test can only directly obtain total passed charge in coulombs, which needs to be converted into the chloride diffusion coefficient by the method proposed by Luping and Nelson [29]. Chemical analysis of chloride concentration (profile) determination is usually combined with the bulk diffusion test introduced in NT Build 443 [30] or ASTM C1556 [31], which is also adopted for chloride resistance determination. In addition, the chloride concentration profile can be obtained by natural diffusion tests, offering a flexible simulation method that mimics the actual field environment closely [32,33,34,35]. Moreover, the chloride profiles obtained by in situ RC structures are also capable of chloride resistance determination [4,20,36,37,38,39].

## 3. Chloride Resistance of Surface-Coated Concrete

This section reviews the experimental and theoretical research on chloride ingress of surface-coated concrete in terms of various coating material categories, including inorganic, organic, and organic–inorganic composite coatings. By comparing the results of different studies, the improvement efficacy in chloride resistance is analyzed and compared emphatically.

It is worth mentioning that for coatings with the same category, the protection mechanisms may differ from each other.

### 3.1. Inorganic Coatings

Inorganic coatings typically exhibit superior weather resistance and chemical resistance, and can withstand ultraviolet light, temperature fluctuations, pH changes, and humidity, offering more stable performance in harsh environmental conditions. Furthermore, inorganic coatings demonstrate better stability and are less prone to decomposition, especially in the case of fire. In this section, we mainly review the studies on inorganic coatings. Table 1 lists chloride diffusion coefficients of concrete coated with various kinds of inorganic materials.

**Figure 3:**
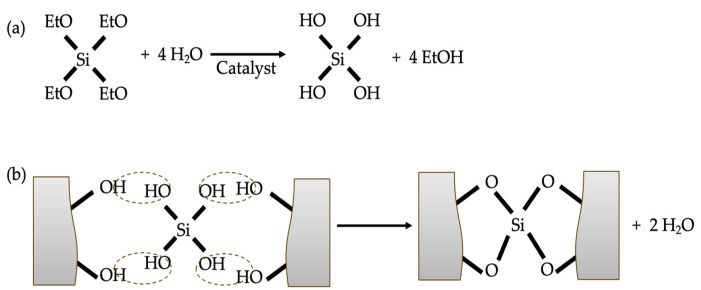
The reaction of ethyl silicate coating with concrete: (**a**) hydrolysis; (**b**) condensation.

Silicate coating, a typical inorganic coating, has been studied by some researchers in terms of both mechanism and specific performance. The precise mechanism through which silicate enhances concrete performance remains uncertain. The silicates act as penetrants/pore blockers due to the generation of SiO_2_ precipitate [40]. In the study of Higgins [41], it is stated that the reaction between concrete components, i.e., water, calcium hydroxide and the silicates, generate calcium silicate hydrate gel (C-S-H). Another theory suggests that the silicate gel generated can fill the pores in concrete, thereby improving its performance [40]. The specific mechanisms of application may vary from case to case. The study of Moon et al. [5] investigated the chloride resistance of mortar coated with a calcium–silicate compound by steady-state migration test. The results indicated the calcium–silicate compound decreased the steady-state chloride diffusion coefficient by 87% and 72% for high strength and low strength, respectively. The analysis results of mercury intrusion porosimetry (MIP) and scanning electron microscopy (SEM) demonstrated that the hydration of the main component in the coating, calcium–silicate, generated water-insoluble silicate compounds, which fill the pores as micro-fillers and form a very dense film on the surface of the mortar. The study of Thompson et al. [40] found that the aqueous sodium silicate coating decreased the electric flux by 45% at 38.6% liquid and 32% at 37.6% liquid. Their studies also indicated that the concentration of solution affected the improvement efficacy. Franzoni et al. [42] compared the chloride resistance of concrete coated with an aqueous sodium silicate solution and an ethyl silicate solution. Their research indicated that sodium silicate exhibited limited effectiveness in protecting against chloride penetration, regardless of whether it was combined with nano SiO_2_. SEM-EDS (energy-dispersive X-ray spectroscopy) analysis found that the sodium silicate-coated concrete surface showed deposited layers, which were detached and exhibited some visible cracking. Conversely, the ethyl silicate coating was compact and less detached, which can be attributed to the consolidating effect of the generated amorphous silica gel in the concrete pores (Figure 3). The study of Pigino et al. [43] showed that ethyl silicate coating decreased the chloride diffusion coefficients of concrete by 98.5% (from 20.4 × 10^−12^ m^2^/s to 0.3 × 10^−12^ m^2^/s) when the *w*/*c* is 0.65 and by 98.6% (from 7.6 × 10^−12^ m^2^/s to 0.1 × 10^−12^ m^2^/s) when the *w*/*c* is 0.45. Sandrolini et al. [44] stated that the superior chloride resistance of ethyl silicate coating can be attributed to (i) the excellent impregnation ability (3–5 mm depth) due to its viscosity being relatively low and its monomer size relatively small; and (ii) the pozzolanic reaction with calcium hydroxide. In addition, ethyl silicate coatings have broad application potential due to their emission of only volatile and non-destructive by-products during the hardening process [42].

**Figure 4:**
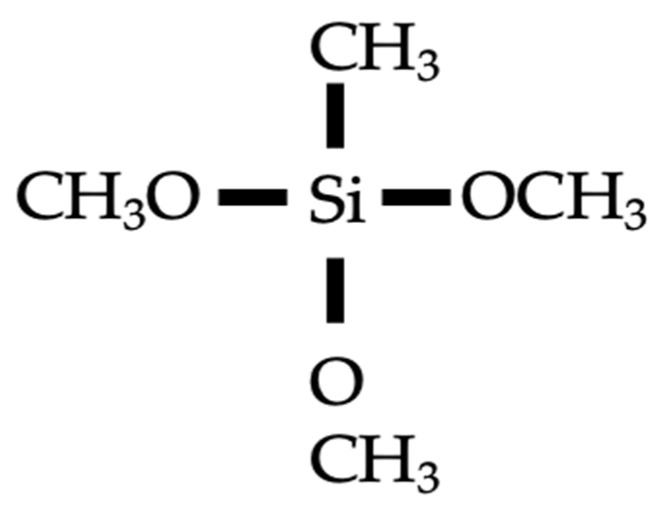
Molecular structure of typical silane.

Silane coatings refer to coatings prepared based on silane compounds, i.e., alkoxy and alkyl silanes, a typical molecular structures of which is shown in Figure 4. The alkoxy groups can react with surface hydration products to generate a silicon oxide layer. The alkylic groups possess organic properties, usually highly hydrophilic, making water molecules tend to form droplets on surfaces and slide off quickly, rather than adsorbing into the surface. This property allows the silicon oxide layer to prevent moisture penetration and surface moisture to a certain extent, helping to protect the concrete under the coating from water erosion and moisture erosion. Medeiros and Helene [6] studied the effectiveness of silane coating on improving chloride resistance. The results indicated that the silane dispersed in the water and solvent decreased the chloride diffusivity of saturated concrete by 9% and 17%, respectively. A similar minor reduction magnitude was reported in another study, which was 11% and 17% [45]. The study of Nanukuttan et al. [36] investigated the chloride penetration of silane-coated concrete subjected to a natural environment for up to 7 years. The diffusion coefficients obtained by Fick’s second law fitting were similar for coated and uncoated conditions. However, in the study of Sakr and Bassuoni [46], the silane effectively improved the chloride resistance of concrete. For example, the chloride diffusion coefficient of coated concreted decreased from 97.28 × 10^–12^ m^2^/s to 11.50 × 10^–12^ m^2^/s (88% reduction) when the *w*/*c* ratio was 0.6. Nevertheless, results from their studies indicated that in terms of improvement in resistance to physical salt attack or frost attack, silane coating was ineffective. The incorporation of nanomaterials, i.e., nano-silica or nano-clay, significantly improved the performance of silane-based composite coating. For example, the chloride diffusion coefficients further decreased by 58%, 58%, 38% and 2% compared with those of single silane-coated concrete when incorporating 5% and 10% dosages of nano-clay and nano-silica, respectively [46]. The improvement efficacy of nano-clay was superior to that of nano-silica, which may be due to the fact that the smaller nano-silica particles are more likely to aggregate and disperse less effectively. Similarly, Ibrahim et al. [47] reported the chloride resistance improvement efficacy of nano-silica and nano-calcium carbonate-modified silane coating. The results indicated that the passing charges decreased by 32% for single silane coating compared to a control group. Compared with single silane coating, the incorporation of a 2.5% dosage of nano-silica showed an 80% decreased passing charge value and 76% for nano-calcium carbonate with the same dosage. In addition, Gu et al. [48] proposed a superhydrophobic coatings based on nano-silica-modified isobutyltriethoxysilane. The superhydrophobic cellular coating was sprayed as the top layer of the coating. The results from rapid chloride migration tests demonstrated that this coating showed superior chloride resistance. The study of Schueremans et al. [49] demonstrated the superior long-term effectiveness of silane coatings. Their findings from on-site experiments concluded that the protection system was still effective even after 12 years.

Geopolymer is an inorganic polymer material characterized by the formation of a three-dimensional network structure through the biochemical reaction of alkaline activators and silica–aluminum skeleton raw materials (such as metallurgical residues, volcanic ash, etc.) at low temperatures. Compared to traditional cement-based materials, geopolymers do not require the use of cement as a cementing material, thus significantly reducing carbon emissions. Geopolymer has excellent mechanical properties, fire resistance, chemical stability and corrosion resistance, so it is used as a new inorganic coating material. The study of Zhuang et al. [50] fabricated a compound geopolymer coating composed of granulated blast furnace slag and metakaolin. Their studies found that this coating showed optimal properties when the content of granulated blast furnace slag was 10% at a liquid/solid ratio of 0.60 mL/g. In another published article [51], they investigated the microstructure and anticorrosion mechanism of this geopolymer coating. The reaction products between the geopolymer slurry and the concrete components were compact and the average open pore size was less than 15 nm. Another typical geopolymer is alkali-activated binder. The study of Phiangphimai et al. [18] fabricated an alkali-activated cement powder composed of geopolymer powder, Portland cement and silica fume. They found the chloride diffusion coefficient of coated concrete decreased by 98% when the ratio of sodium silicate to sodium hydroxide was 1.0 and 97% when the ratio of sodium silicate to sodium hydroxide was 2.0.

Currently, graphene oxide, a promising nanomaterial, has increasingly garnered attention in research circles. At present, most practices are to add graphene oxide to cement slurry to improve its performance. Although this approach can improve the mechanical performance [52,53,54] and durability [55] of cement pastes, the efficacy is far from satisfactory and the fresh mixing performance is adversely affected [56,57]. Antolín-Rodríguez et al. [58] investigated the effectiveness of graphene oxide as a surface treatment technique on improving the chloride penetration of coated concrete. Their results showed the chloride diffusion coefficient determined by rapid chloride migration tests decreased by 4%, 18.9%, 36.6%, 40.3% and 51% when the graphene oxide contents were 26.2, 52.4, 78.6, 104.8, and 131.1 μg/cm^2^. In the study of Antolín-Rodríguez [59], the SEM analysis demonstrated that the microstructure of the surface was densified due to the facilitation effect of graphene on the hydration process. In another study of Antolín-Rodríguez [60], the amount of graphene oxide coating significantly showed a direct correlation with the protection efficacy. However, it is essential to determine an optimal level of graphene oxide content that strikes a balance between efficacy and economy in the treatment process.

**Table 1:** Chloride diffusivity of concrete coated with inorganic coating materials.

Ref.	Coating Type	*w*/*c*	Chloride Diffusion Coefficient (D = 10^−12^ m^2^/s)/Electric Flux (C)	Test Method	Control Group	Coating Process
[43]	Calcium–silicate compound M1	0.485	0.3D	SMM	2.3D	Spraying
[43]	Calcium–silicate compound-M2.45	0.485	1.8D	SMM	6.5D	Spraying
[40]	Aqueous sodium silicate—N	0.48	8993C	RCPT	13219C	Brushing
[40]	Aqueous sodium silicate—OW	0.48	7238C	RCPT		Brushing
[44]	Ethyl silicate	0.65	0.3D	RCMT	20.4D	Brushing
[44]	Ethyl silicate	0.45	0.1D	RCMT	7.6D	Brushing
[18]	Alkali-activated/cement powder -S/H ratio of 1.0	0.5	0.081D	RCMT	6.212D	-
[18]	Alkali-activated/cement powder -S/H ratio of 2.0	0.5	0.192D	RCMT	6.212D	-
[61]	Graphene oxide (26.2 μg/cm^2^)	0.5	1.52D	SSM	1.59D	Spraying
			5.84D	RCMT	6.08D	
[61]	Graphene oxide (52.4 μg/cm^2^)	0.5	1.36D	SSM	1.59D	Spraying
			5.65D	RCMT	6.08D	
[61]	Graphene oxide (78.6 μg/cm^2^)	0.5	1.03D	SSM	1.59D	Spraying
			3.85D	RCMT	6.08D	
[61]	Graphene oxide (104.8 μg/cm^2^)	0.5	0.55D	SSM	1.59D	Spraying
			3.63D	RCMT	6.08D	
[61]	Graphene oxide (131.1 μg/cm^2^)	0.5	0.39D	SSM	1.59D	Spraying
			2.97D	RCMT	6.08D	
[49]	Silane	0.6	11.5D	RCPT	97.28D	Brushing
			1788C		OVF	
[49]	silane/nano-clay (5%)	0.6	4.88D	RCPT	97.28D	Brushing
			832C		OVF	
[49]	silane/nano-clay (10%)	0.6	4.86D	RCPT	97.28D	Brushing
			785C		OVF	
[49]	silane/nano-silica (5%)	0.6	7.14D	RCPT	97.28D	Brushing
			1012C		OVF	
[49]	silane/nano-silica (10%)	0.6	11.30D	RCPT	97.28D	Brushing
			1530C		OVF	
[49]	Silane	0.4	5.33D	RCPT	20.93D	Brushing
			963C		4015C	
[49]	silane/nano-clay (5%)	0.4	3.13D	RCPT	20.93D	Brushing
			425C		4015C	
[49]	silane/nano-clay (10%)	0.4	3.64D	RCPT	20.93D	Brushing
			488C		4015C	
[49]	silane/nano-silica (5%)	0.4	3.29D	RCPT	20.93D	Brushing
			466C		4015C	
[49]	silane/nano-silica (10%)	0.4	4.44D	RCPT	20.93D	Brushing
			609C		4015C	
[6]	Silane/siloxane dispersed in water	0.52	3.13D	RCPT	3.45D	Soaking
[6]	Silane/siloxane dispersed in solvent	0.52	2.85D	RCPT	3.45D	Soaking
[50]	Silane	0.6	268C	RCPT	OVF	Brushing
[50]	silane/nano-clay (2.5%)	0.6	641C	RCPT	OVF	Brushing
[50]	silane/nano-clay (5%)	0.6	750C	RCPT	OVF	Brushing
[50]	silane/nano-CaCO3 (2.5%)	0.6	488C	RCPT	OVF	Brushing
[50]	silane/nano-CaCO3 (5%)	0.6	726C	RCPT	OVF	Brushing
[50]	Silane	0.4	211C	RCPT	2326C	Brushing
[50]	silane/nano-clay (2.5%)	0.4	307C	RCPT	2326C	Brushing
[50]	silane/nano-clay (5%)	0.4	336C	RCPT	2326C	Brushing
[50]	silane/nano-CaCO3 (2.5%)	0.4	378C	RCPT	2326C	Brushing
[50]	silane/nano-CaCO3 (5%)	0.4	596C	RCPT	2326C	Brushing
[10]	silane	0.6	229C	RCPT	2745C	Brushing
[62]	Alkyl alkoxysilane	0.45	8.7D	FT	9.0D	/

Note: (i) M1 denotes the ratio of sand to cement is 1, M2.45 denotes the ratio of sand to cement is 2.45; (ii) N denotes the aqueous sodium silicate had 37.6% solids, OW denotes the aqueous sodium silicate had 38.6% solids; (iii) S/H ratio denotes the ratio of sodium silicate to sodium hydroxide. (iv) SMM denotes the steady-state migration method; RCPT denotes rapid chloride permeability test; RCMT denotes rapid chloride migration test. (v) OVF denotes overflow, which means the RCPT process is terminated before 6 h.

### 3.2. Organic Coatings

In this study, coatings made of organic chemicals are classified as organic coatings. Table 2 lists the chloride diffusion coefficients of concrete coated with various kinds of organic materials.

**Table 2:** Chloride diffusivity of concrete coated with organic coating materials.

Ref.	Coating Type	*w*/*c*	Chloride Diffusion Coefficient (D = 10^−12^ m^2^/s)/Electric Flux (C)	Test Method	Control Group	Coating Process
[63]	Acrylic coating	0.6	6.53D	RCMT	14.4D	/
[63]	Epoxy coating	0.6	0D	RCMT	14.4D	/
[63]	Acrylic coating	0.4	3.86D	RCMT	6.9D	/
[63]	Epoxy coating	0.4	0D	RCMT	6.9D	/
[64]	Acrylic coating, AC1	0.45	2.08D	IT	19.18D	/
[64]	Acrylic coating, AC2	0.45	3.49D	IT	19.18D	/
[64]	Polymer emulsion coating, PE1	0.45	8.40D	IT	19.18D	/
[64]	Polymer emulsion coating, PE2	0.45	15.97D	IT	19.18D	/
[64]	Epoxy coating, EP1	0.45	7.67D	IT	19.18D	/
[64]	Epoxy coating, EP2	0.45	2.59D	IT	19.18D	/
[64]	Polyurethane coating, PU1	0.45	1.83D	IT	19.18D	/
[64]	Polyurethane coating, PU2	0.45	0.70D	IT	19.18D	/
[64]	Chlorinated rubber coating, CR1	0.45	9.56D	IT	19.18D	/
[64]	Chlorinated rubber coating, CR2	0.45	8.40D	IT	19.18D	/
[62]	Acrylic sealant	0.45	11.2D	FT	9.0D	Brushing
[62]	Polyurethane sealant	0.45	8.8D	FT	9.0D	Brushing
[62]	Acrylic coating	0.45	9.0D	FT	9.0D	Brushing
[62]	Polyurethane coating	0.45	4.7D	FT	9.0D	Brushing
[65]	Polyurethane–SiO_2_ (0%)	0.6	2110C	RCPT	/	Spraying
[65]	Polyurethane–SiO_2_ (1%)	0.6	1780C	RCPT	/	Spraying
[65]	Polyurethane–SiO_2_ (2%)	0.6	1520C	RCPT	/	Spraying
[65]	Polyurethane–SiO_2_ (3%)	0.6	1470C	RCPT	/	Spraying
[65]	Epoxy resin–SiO_2_ (0%)	0.6	610C	RCPT	/	Spraying
[65]	Epoxy resin–SiO_2_ (1%)	0.6	450C	RCPT	/	Spraying
[65]	Epoxy resin–SiO_2_ (2%)	0.6	390C	RCPT	/	Spraying
[65]	Epoxy resin–SiO_2_ (3%)	0.6	430C	RCPT	/	Spraying
[65]	Polyurethane–TiO_2_ (0%)	0.6	2110C	RCPT	/	Spraying
[65]	Polyurethane–TiO_2_ (1%)	0.6	1670C	RCPT	/	Spraying
[65]	Polyurethane–TiO_2_ (2%)	0.6	1480C	RCPT	/	Spraying
[65]	Polyurethane–TiO_2_ (3%)	0.6	1720C	RCPT	/	Spraying
[65]	Epoxy resin–TiO_2_ (0%)	0.6	610C	RCPT	/	Spraying
[65]	Epoxy resin–TiO_2_ (1%)	0.6	460C	RCPT	/	Spraying
[65]	Epoxy resin–TiO_2_ (2%)	0.6	430C	RCPT	/	Spraying
[65]	Epoxy resin–TiO_2_ (3%)	0.6	490C	RCPT	/	Spraying
[66]	Epoxy coating—submicron/nano carbon (0%)	0.45	102D	PT	134D	Soaking
[66]	Epoxy coating—submicron/nano carbon (0.25%)	0.45	98D	PT	134D	Soaking
[66]	Epoxy coating—submicron/nano carbon (0.5%)	0.45	92D	PT	134D	Soaking
[66]	Epoxy coating—submicron/nano carbon (0.75%)	0.45	78D	PT	134D	Soaking
[66]	Epoxy coating—submicron/nano carbon (1.0%)	0.45	70D	PT	134D	Soaking
[67]	Modified acrylic styrene emulsion	0.5	5.944D	RCMT	7.799D	/
[10]	epoxy glass flake paint	0.6	752C	RCPT	2745C	Brushing
[10]	Polyurethane paint	0.6	550C	RCPT	2745C	Brushing
[46]	Poly(methyl methacrylate) (PMMA)	0.6	17.5D	RCPT	97.28D	Brushing
			2981C		OVF	
[46]	PMMA/nano-clay (5%)	0.6	4.88D	RCPT	97.28D	Brushing
			832C		OVF	
[46]	PMMA/nano-clay (10%)	0.6	4.86D	RCPT	97.28D	Brushing
			785C		OVF	
[46]	PMMA/nano-silica (5%)	0.6	7.14D	RCPT	97.28D	Brushing
			1012C		OVF	
[46]	PMMA/nano-silica (10%)	0.6	11.30D	RCPT	97.28D	Brushing
			1530C		OVF	
[46]	Poly (methyl methacrylate) (PMMA)	0.4	11.87D	RCPT	20.93D	Brushing
			1785C		4015C	
[46]	PMMA/nano-clay (5%)	0.4	4.00D	RCPT	20.93D	Brushing
			569C		4015C	
[46]	PMMA/nano-clay (10%)	0.4	5.78D	RCPT	20.93D	Brushing
			826C		4015C	
[46]	PMMA/nano-silica (5%)	0.4	4.70D	RCPT	20.93D	Brushing
			761C		4015C	
[46]	PMMA/nano-silica (10%)	0.4	10.38D	RCPT	20.93D	Brushing
			1542C		4015C	
[47]	Vinyl ester	0.6	4485C	RCPT	OVF	Brushing
[47]	Vinyl ester/nano-clay (2.5%)	0.6	63C	RCPT	OVF	Brushing
[47]	Vinyl ester/nano-clay (5%)	0.6	153C	RCPT	OVF	Brushing
[47]	Vinyl ester/nano-CaCO_3_ (2.5%)	0.6	74C	RCPT	OVF	Brushing
[47]	Vinyl ester/nano-CaCO_3_ (5%)	0.6	44C	RCPT	OVF	Brushing
[47]	Vinyl ester	0.4	1576C	RCPT	2326C	Brushing
[47]	Vinyl ester/nano-clay (2.5%)	0.4	10C	RCPT	2326C	Brushing
[47]	Vinyl ester/nano-clay (5%)	0.4	66C	RCPT	2326C	Brushing
[47]	Vinyl ester/nano-CaCO_3_ (2.5%)	0.4	34C	RCPT	2326C	Brushing
[47]	Vinyl ester/nano-CaCO_3_ (5%)	0.4	23C	RCPT	2326C	Brushing
[68]	TA coating (Fe(III))—self-polymerization	0.5	4.97D	RCPT	5.2D	Soaking
			1163C		1617C	
[68]	TA coating (Fe(III))—one step method	0.5	4.18D	RCPT	5.2D	Soaking
			1114C		1617C	
[68]	TA coating (Fe(III))—multi-step method	0.5	4.13D	RCPT	5.2D	Soaking
			1249C		1617C	
[9]	TA-APTES-HDTMS (1%)	0.5	779C	RCPT	1657.81C	Soaking
[9]	TA-APTES-HDTMS (3%)	0.5	554C	RCPT	1657.81C	Soaking
[9]	TA-APTES-HDTMS (5%)	0.5	494C	RCPT	1657.81C	Soaking
[9]	TA-APTES-HDTMS (7%)	0.5	429C	RCPT	1657.81C	Soaking
[9]	TA-APTES-HDTMS (10%)	0.5	391C	RCPT	1657.81C	Soaking
[9]	TA-APTES-HDTMS (15%)	0.5	367C	RCPT	1657.81C	Soaking
[69]	D-CO2-monoethanolamine (MEA) (10%)	0.35	10.7D	RCPT	13.3D	/
			296.09C		339.07C	
[69]	D-CO_2_-monoethanolamine (MEA) (30%)	0.35	9.1D	RCPT	13.3D	/
			285.16C		339.07C	
[69]	F-CO_2_-monoethanolamine (MEA) (10%)	0.35	13.6D	RCPT	13.3D	/
			336.30C		339.07C	
[69]	F-CO_2_-monoethanolamine (MEA) (30%)	0.35	13.5D	RCPT	13.3D	/
			336.00C		339.07C	
[69]	D-CO_2_-monoethanolamine (MEA) (10%)	0.4	13.0D	RCPT	18.1D	/
			339.10C		458.9C	
[69]	D-CO_2_-monoethanolamine (MEA) (30%)	0.4	12.4D	RCPT	18.1D	/
			293.77C		458.9C	
[69]	D-CO_2_-monoethanolamine (MEA) (10%)	0.5	19.0D	RCPT	20.4D	/
			440.86C		539.43C	
[69]	D-CO_2_-monoethanolamine (MEA) (30%)	0.5	18.2D	RCPT	20.4D 539.43D	/

**Figure 5:**
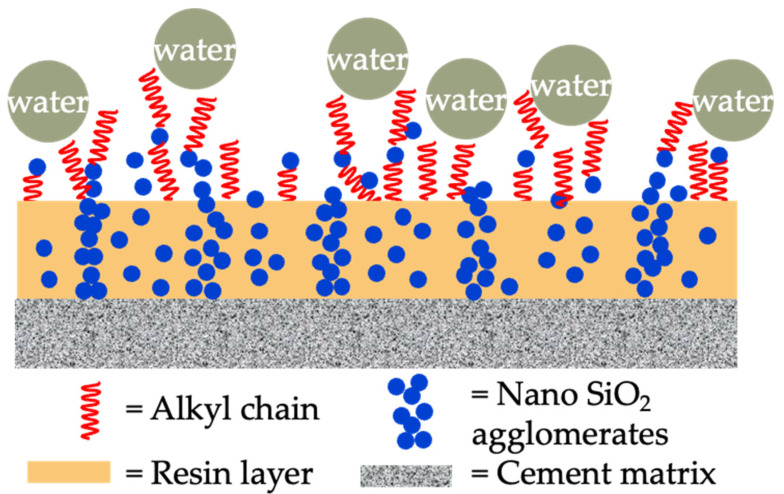
Microstructure model of organic coating modified by nanomaterials.

Conventional organic coating refers to coatings made from organic polymers, including resins, solvents, and additives. Common organic polymers are polyester resin, polyacrylic resin, polyurethane resin, and so on. Aguiar et al. [63] evaluated the effect of acrylic and epoxy polymeric coatings on chloride penetration resistance of concrete. The results indicated that use of both acrylic and epoxy coatings contributed to a significantly decreased chloride diffusion coefficient. In the presence of epoxy resin coating, the diffusion coefficient of concrete even achieved null. Almusallam et al. [64] investigated five generic types of polymeric coatings. The acrylic, polymer emulsion, epoxy polyurethane, and chlorinated coating decreased the chloride diffusion coefficients by 89%/82%, 56%/17%, 60%/86%, 90%/96%, and 50%/56%, respectively. The values before and after the slash “/” represent that the same coating comes from different manufacturers. This suggests that the determination of coating efficacy should rely on preliminary tests rather on the types alone. Shi et al. [67] investigated the improvement effect of a modified styrene–acrylic emulsion on chloride diffusion of coated concrete. Compared with a control group, the penetration depth decreased from 18.5 mm to 13.25 mm, and the diffusion coefficient decreased by 24% with polymer thickness of 70 μm. Li et al. [10] investigated and compared the chloride resistance and service life prediction of epoxy glass-flake paint, polyurethane paint, cement-based permeable crystallization waterproof coating, and silane-based water-repellent coating. The results indicated that the corresponding coulomb electric flux decreases were 92%, 90%, 73% and 80%. Although the chloride resistance of polymer coatings was superior to silane coatings, the aging test results indicated that weather fastness of silane was more superior. The excellent weather resistance of silane has also been reported in the study of Christodoulou et al. [20], which stated that even after 20 years, the silane impregnation still exhibited a residual hydrophobic effect. Qu et al. [65] studied the improvement effect of nanomaterial-modified water-based polyurethane and epoxy resin coatings on chloride resistance of coated concrete. The addition of nano-SiO_2_ and nano-TiO_2_ improved the coulomb electric flux of neat resin coated concrete by 51–74% and 24–27%, respectively. In addition, the nanomaterials also improved the anti-aging capability of water-based organic coatings. The long-term anti-aging capability improvement effect of nano-TiO_2_ was more significant than that of nano-SiO_2_. Basha et al. [66] investigated the improvement effect of epoxy coating modified by submicron/nano-carbon obtained from the waste material generated from heavy fuel oil ash combustion. The results indicated the submicron/nano-carbon with dosage of 1% performed best in increasing the chloride resistance of coated concrete (with 66% reduction). In brief, nanomaterials show promise in enhancing the performance of conventional organic coatings by effectively filling micropores and the hydrophobic effect, as shown in Figure 5.

Although conventional organic coatings show predominance in chloride resistance improvement, their defects, i.e., bringing toxic by-products during the production process and releasing voltaic organic compounds during the coating process, are also troublesome. Thus, the research community have tried to fabricate novel environmentally friendly anti-corrosion coatings. Inspired by the robust adhesion between mussels and rock, a bionic coating for mussels based on polyphenol material tannic acid (TA) for concrete protection was proposed by Xiao et al. [68]. The results indicated the TA-trivalent iron cation (Fe(III)) coating showed comparable chloride resistance improvement effect to conventional organic coatings. On this basis, Liao et al. [9] performed hydrophobic modification of the TA coating, seeking to further improve the performance of the coating. They utilized the organosilicon compound hexadecyltrimethoxysilane (HDTMS) to modify a TA-aminopropyltriethoxysilane (APTES) coating and successfully fabricated a hydrophobic nanocomposite coating. They also investigated the effect of TA, APTES, and HDTMS concentration on coating performance and fabricated an optimal TA–APTES–HDTMS coating. The coulomb electric flux was decreased by more than 70% after coating. Additionally, methyl methacrylate and vinyl ester, which are not adequately effective to protect concrete from chloride erosion alone, can also be used in combination with nanomaterials for hydrophobic modification and performance improvement. In the study of Sakr and Bassuoni [46], the chloride resistance of sole and nano-clay- or nano-silica-modified methyl methacrylate-coated concrete was investigated. The results indicated that the incorporation of 5% nano-clay, 10% nano-clay, 5% nano-silica, and 10% nano-silica decreased the diffusion coefficients by 66%/72%, 51%/72%, 60%/59%, and 12%/35%, respectively. The values before and after the slash “/” represent substrate concretes with *w*/*c* of 0.4 and 0.6. In the study of Ibrahim et al. [47], they investigated the chloride resistance of sole and nano-clay- or nano-CaCO_3_-modified vinyl ester-coated concrete. The results indicated that both 2.5% and 5% nano-clay or nano-CaCO_3_ incorporated vinyl ester-coated concrete showed almost 100% decreased coulomb electric flux compared with neat vinyl ester-coated concrete. In addition, Kong et al. [70] synthesized a sustainable superhydrophobic concrete coating by micro-diatomaceous earth, nano-Al_2_O_3_, and stearic acid, which exhibited excellent anti-corrosion performance on electrochemical corrosion tests. Luo et al. [71] successfully fabricated a robust superhydrophobic coating with myristic acid and ZrO_2_ particles. However, the chloride resistance of this novel hydrophobic coating has not been investigated.

In the study of Han et al. [69], the chloride resistance improvement effect of CO_2_ dissolved monoethanolamine (MEA) on coated concrete was investigated. The HCO_3_^−^ and CO_3_^2−^ in CO_2_-dissolved MEA solution can react with the hydration product, i.e., Ca (OH)_2_, C_3_S, and C_2_S, to generate calcium carbonate precipitation, which can act as filler to densify the surface of the concrete substrate. The test results indicated that the performance of this coating was affected by various factors, i.e., MEA concentration, the quality of the substrate, coating time and pre-condition method. The treatment of this coating with a 10% dosage of MEA on concrete with 0.4 *w*/*c* ratio showed optimal chloride resistance (with 31.5% reduction).

### 3.3. Organic–Inorganic Composite Coatings

Organic–inorganic composite coating refers to the coating system that contains both organic and inorganic components. It is worth noting that the composite coatings made by combining an organic resin (such as a polymer) with an inorganic material (such as silica, zinc oxide, etc.) or inorganic particles (such as nanoparticles) belong to this type, which have been detailed in previous sections. In this section, the polymer-modified cementitious/geopolymer coatings are mainly focused on. Table 3 lists the chloride diffusion coefficients of concrete coated with various kinds of organic–inorganic composite coatings.

Zhang et al. [62] analyzed and compared the interfacial chloride concentration and pseudo-chloride diffusion coefficient of acrylic-modified cementitious coating and other pure polymer coatings. The results showed that the interfacial chloride concentration of composite coating-treated concrete was far lower than that of untreated concrete, but higher than that of sole acrylic- or polyurethane-treated concrete. The same was found for the pseudo-diffusion coefficients. Diamanti et al. [72] investigated the influence of polymer–cement ratio on the improvement efficiency of acrylic-modified ordinary Portland cement coating in chloride resistance of coated concrete. The results indicated that the coating porosity decreased with increasing polymer content, and thus the effectiveness of the barrier to chloride ingress was increased. Liang et al. [73] investigated the influence of fiber introduction on the protection effectiveness of silicone-modified polyacrylate modified calcium sulfoaluminate cement coating. The results indicated the polypropylene fiber showed obvious effects in improving the tensile strength, bonding strength, wear resistance, and anti-UV aging resistance, and the chloride penetration resistance was significantly increased (with 49% reduction rate compared to uncoated concrete). Zhang et al. [74] proposed a waterborne epoxy resin and silane coupling agent-modified metakaolin-based geopolymer coating, which exhibited excellent performance in shrinkage reduction, adhesion, anti-abrasion, and chloride corrosion resistance. However, the specific chloride resistance has not been investigated yet.

**Table 3:** Chloride diffusivity of concrete coated with organic–inorganic composite coating materials.

Ref.	Coating Type	*w*/*c*	Chloride Diffusion Coefficient (D = 10^−12^ m^2^/s)/Electric Flux (C)	Test Method	Control Group	Coating Process
[10]	Cement-based permeable crystallization waterproof coating	0.6	279C	RCPT	2745C	Brushing
[62]	Two-component acrylic-modified cementitious coating	0.45	4.7D	FT	9.0D	Brushing
[72]	Acrylic–cementitious coating (PC ratio of 0.35)	0.65	0.50D	SSM	8.03D	/
[72]	Acrylic–cementitious coating (PC ratio of 0.55)	0.65	0.28D	SSM	8.03D	/
[72]	Acrylic–cementitious coating (PC ratio of 0.35)	0.5	0.13D	SSM	2.41D	/
[72]	Acrylic–cementitious coating (PC ratio of 0.55)	0.5	0.15D	SSM	2.41D	/
[73]	Silicone-modified polyacrylate-modified cement-based coating	/	2665.42C	RCPT	4054.00C	/
[73]	silicone-modified polyacrylate-modified cement-based coating incorporating polypropylene fiber	/	2070.64C	RCPT	4054.00C	/

## 4. Modeling Chloride Penetration into Surface-Coated Concrete

**Figure 6:**
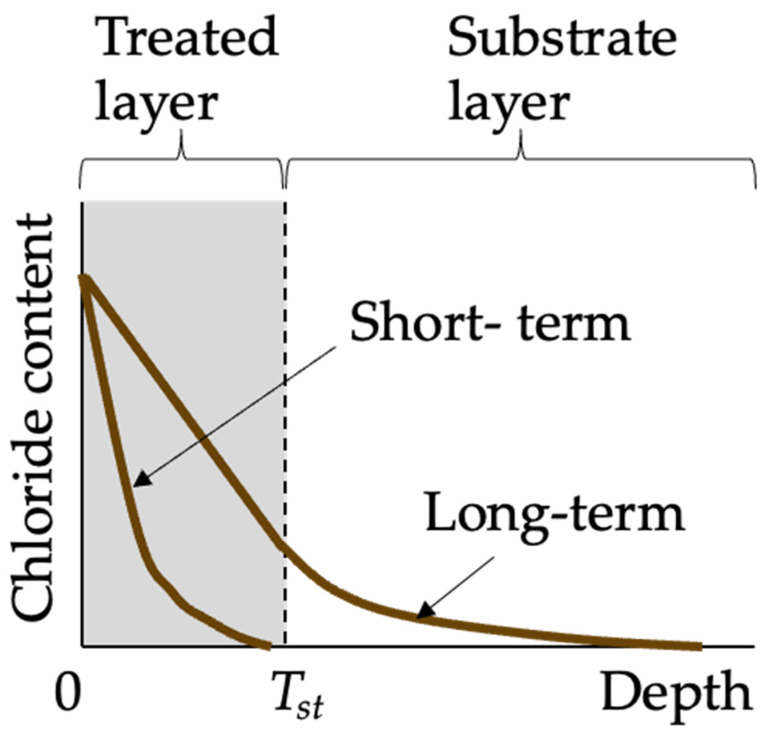
Chloride profiles in coated concrete.

The goal of modeling chloride penetration into concrete is to predict chloride concentration profiles, which are crucial for determining the chloride concentration at the reinforcement depth and thus forecasting the service life of the concrete structure. Chloride transport in coated concrete is influenced by several factors, including both the properties of the concrete substrate and coating. Current studies on chloride transport modeling typically treat coated concrete as a bilayer material [75] consisting of the treated layer and the substrate layer, as illustrated in Figure 6. In the case of coatings with a film-forming mechanism, the treated layer represents the additional physical layer formed by the coating. For coatings that function through a pore-blocking mechanism, the treated layer corresponds to the concrete surface layer that has been impregnated with coating materials. However, for coatings that employ a pore-liner mechanism, there is currently a lack of specific chloride transport modeling approaches.

**Figure 7:**
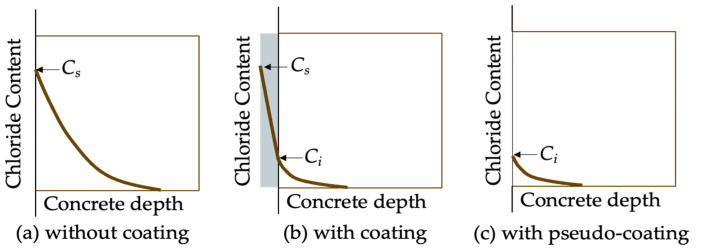
Schematic diagram of pseudo-diffusion model.

Compared to the chloride profiles in uncoated concrete, the chloride profiles in coated concrete often appear as a piecewise function, as illustrated in Figure 7. This is due to the differing diffusion coefficients between the treated layer and the substrate layer. Moradllo et al. [36,37] fitted the analytical solution of Fick’s second law (Equation (1)) directly to the chloride profiles of untreated substrate concrete, without accounting for the chloride profiles of the surface-treated layer. The obtained surface chloride concentration is termed interfacial chloride concentration *C_i_*; and the obtained diffusion coefficient *D_a_* is termed pseudo-diffusion coefficient [62]. In the study of Moradllo et al. [36,37], the pseudo-diffusion coefficient was firstly smaller than the true diffusion coefficient of the substrate concrete *D*_0_, and then increased to over *D*_0_ with the degradation of coating materials. To put it simply, both *C_i_* and pseudo-diffusion coefficients are time-dependent, which violates the premise for the existence of the simple closed-form solution (Equation (1)), that is, both *C_i_* and pseudo-diffusion coefficients are assumed to be constant. For remedying this limitation, Petcherdchoo [17] proposed a time-dependent pseudo-coating model (Equation (2)). This model was validated by comparison with experimental data:

(1) $$C\left(x,t\right)=C_{i}\left[1-erf\left(\frac{x}{2\sqrt{D_{a}t}}\right)\right]$$

(2) $$\begin{array}{l} C\left(x,t\right)=C_{0,t}\left[erfc\left(\frac{x}{2\sqrt{D_{a}t}}\right)\right]+k_{l}t\left[\left(1+\frac{x^{2}}{2D_{a}t}\right)erfc\left(\frac{x}{2\sqrt{D_{a}t}}\right)-\left(\frac{x}{\sqrt{\pi D_{a}t}}\right)e^{-\frac{x^{2}}{4D_{a}t}}\right]\\ D_{a}=\frac{D_{28}}{1-m}\left[{\left(1+\frac{28}{365t}\right)}^{1-m}-{\left(\frac{28}{365t}\right)}^{1-m}\right]\cdot {\left(\frac{28}{365t}\right)}^{m} \end{array}$$

where *D_a_* denotes the obtained apparent diffusion coefficient; *C_i_* denotes the interfacial chloride concentration; *t* denotes exposure time; *D*_28_ denotes the initial chloride diffusion at 28 days’ curing; *m* denotes the aging coefficient; *C*_0,*l*_ denotes the initial chloride content; and *k_l_* is the fitting constant.

The pseudo-diffusion coefficient is affected by multiple factors and mechanisms, such as the diffusion properties of both treated and substrate layers and chloride binding. In some cases, integrating multiple factors into a one-parameter pseudo-diffusion coefficient may make decisions more efficient. At the same time, some information may be lost or blurred, because the uniqueness of specific factors may be consolidated into an average or composite value, making decisions based on this integrated value unreasonable and unscientific. To remedy this limitation, Zhang et al. [76] adopted the finite difference method to solve the chloride diffusion model in saturated coated concrete. In the diffusion model (Equations (3)–(6)), the properties of both treated layer and untreated substrate layer are separately considered. It is worth noting that the water-percolated porosity rather than the total porosity of the treated layer is used. It is necessary to use the water-percolated porosity, which contributes to chloride diffusion. For instance, the silane coating may not alter the total porosity, but obviously decreases the water-percolated porosity. In addition, Zhang et al. [76] analyzed the dependence of the pseudo-diffusion coefficient on the chloride diffusion coefficient of treated layer *D_st_*, water-percolated porosity *P_st_*, and the thickness of treated layer *T_st_*. The results indicated that the pseudo-diffusion coefficient decreased with *T_st_*, but increased with *P_st_* and *D_st_*.

(3) $$\frac{\partial C}{\partial t}=\frac{\partial }{\partial x}\left(D\frac{\partial C}{\partial x}\right)$$

The initial condition:

(4) C=0, t=0, x≥0

Boundary conditions:

(5) $$x=\infty ,\;C=0;\;x=0,\;C=C_{0},\;t>0$$

where *C*_0_ denotes the external chloride content.

The continuity conditions in the interface of the treated layer and concrete substrate layer:

(6) $$\begin{array}{l} P_{st}D_{st}{\frac{\partial C}{\partial x}|}_{x=T_{st}}=P_{su}D_{su}{\frac{\partial C}{\partial x}|}_{x=T_{st}}\\ C_{st}=C_{su} \end{array}$$

where *P_st_* denotes water-percolated porosity; *P_su_* denotes the porosity of untreated concrete substrate; and *D_st_* and *D_su_* denote the chloride diffusion coefficient of treated layer and untreated concrete substrate, respectively. *C_st_* and *C_su_* denote the interfacial chloride content; and *T_st_* denotes the thickness of treated layer.

Although the finite difference method can solve the chloride concentration profile, the calculation process is inefficient due to the low thickness of the treatment layer and the high requirement of space division. This limitation is especially prominent in the reliability calculation based on the Monte Carlo method. In response to this, Wang et al. [77] proposed an efficient mathematical method for predicting chloride transport in bilayer materials, that is, a rapid numerical approach. The diffusion equation is usually a partial differential equation with time and space variables. According to the separation-of-variables method, the function of the change in chloride ion concentration with respect to time and space can be written as the product of the part that depends only on space x and the part that depends only on time t, as in Equation (7). The analytical solution in Equation (7) is only valid when the diffusion coefficient is constant. By a step function, the chloride diffusion coefficient can be considered constant at each time step that is small enough. This approximation method allows the realization of rapid calculation, which significantly improves the calculation efficiency [75]:

(7) $$C\left(x,T\right)=C_{0}+\sum_{n=1}^{\infty }A_{n}f_{n}\left(x_{n},\lambda_{n}\right)\exp\left(-\lambda_{n}^{2}t\right)$$

where *A_n_* is a coefficient that can be determined by known conditions; *f_n_* is a function that can be determined by known conditions; $\lambda_{n}$ and is the characteristic root.

## 5. Outlook

Effective coating materials for improving the chloride resistance of concrete have been widely developed and investigated, but more targeted studies are needed to quantify chloride transport behavior in coated concrete in the natural environment. This section presents an outlook for future research based on current studies regarding the chloride resistance of surface-coated concrete. Future research can focus on the following areas.

(i) There is significant disagreement in current research regarding the efficiency of silane-based coatings in enhancing concrete’s resistance to chloride. Further detailed studies are needed, preferably considering the effects of coating processes, coating concentration, and the properties of concrete itself on improvement efficiency.

(ii) Although traditional coatings have demonstrated excellent performance in enhancing concrete’s resistance to chloride, their environmental pollution limits their widespread use. Future work shall focus on developing green, environmentally friendly, safe, efficient, and low-cost multifunctional composite coatings.

(iii) Existing studies indicate that coating processes, sources of coating raw materials, and concentration ratios all influence the effectiveness of coatings in enhancing resistance to chloride. Further research should address this point specifically.

(iv) While current research has investigated the resistance of coatings to chloride, the focus has primarily been on short-term performance and laboratory studies. Long-term field tests should be conducted to gather data on the chloride ion transport behavior of coated concrete under natural climatic conditions.

(v) Current chloride transport modeling of coated concrete only considers diffusion mechanisms or modified parameters based on Fick’s diffusion law to incorporate convective phenomena. The effect of coatings on moisture transport, bound and free chloride conversion, etc., behaviors also need to be considered. Moreover, the change in the treated layer properties with time will obviously affect the interfacial chloride concentration, thus affecting the chloride transport behavior. In future research, an effective model with interpretability, strong predictive ability, and practical application feasibility shall be developed, which can help to predict the service life.

(vi) The determination of the porosity, chloride profiles, and diffusion coefficient of the treated layer is still unclear, for which advanced techniques may be required to address this limitation.

(vii) The application and selection of coating shall consider the sustainability of coated concrete, including material selection, life cycle costs, and environmental impact assessments, to promote its widespread use in engineering practice.

Through the in-depth exploration of the above research directions, the performance and reliability of coated concrete in practical applications can be further improved, and more sustainable and durable solutions can be provided for engineering construction.

## 6. Conclusions

Concrete coatings have increasingly garnered attention for their promising potential in enhancing the chloride resistance of concrete structures, whether they are new or in service. A comprehensive review of reported studies on chloride penetration of coated concrete is presented in this study, including a specific elaboration of protection mechanisms, discussion of chloride resistance enhancement efficiency, summary of chloride transport modeling methodologies, and outlook for future research directions. For protection mechanisms, coatings can serve as continuous compact physical barriers, penetrants, waterproof films, or a combination of these three roles. Special emphasis shall be placed on identifying protective mechanisms, which may significantly influence the effectiveness, deterioration, and methodologies used to quantify chloride transport in the coatings. For chloride resistance enhancement efficiency, this study has thoroughly reviewed and compared the data reported in current studies. Although a great volume of research has been conducted in this field, few studies can provide data for quantitatively analyzing chloride transport behavior, especially for long-term in situ data, which are crucial for practical application. For modeling of chloride transport, the current methodologies can provide a rough quantification of chloride concentration profiles. More accurate physical models shall be developed in future research. Overall, while substantial progress has been made in developing effective coatings and studying the chloride resistance of coated concrete, further research and development in this critical area are necessary to enable broader applications of coatings in marine engineering.
